# Genomics Study of the Exposure Effect of *Gymnodinium catenatum*, a Paralyzing Toxin Producer, on *Crassostrea gigas*' Defense System and Detoxification Genes

**DOI:** 10.1371/journal.pone.0072323

**Published:** 2013-09-10

**Authors:** Norma García-Lagunas, Reyna Romero-Geraldo, Norma Y. Hernández-Saavedra

**Affiliations:** 1 Laboratorio de Genética Molecular, Centro de Investigaciones Biológicas del Noroeste (CIBNOR), La Paz, Baja California Sur, México; 2 Instituto Tecnológico de La Paz, La Paz, Baja California Sur, México; Cinvestav, Mexico

## Abstract

**Background:**

*Crassostrea gigas* accumulates paralytic shellfish toxins (PST) associated with red tide species as *Gymnodinium catenatum*. Previous studies demonstrated bivalves show variable feeding responses to toxic algae at physiological level; recently, only one study has reported biochemical changes in the transcript level of the genes involved in *C. gigas* stress response.

**Principal Findings:**

We found that 24 h feeding on toxic dinoflagellate cells (acute exposure) induced a significant decrease in clearance rate and expression level changes of the genes involved in antioxidant defense (copper/zinc superoxide dismutase, Cu/Zn-SOD), cell detoxification (glutathione S-transferase, GST and cytochrome P450, CPY450), intermediate immune response activation (lipopolysaccharide and beta glucan binding protein, LGBP), and stress responses (glutamine synthetase, GS) in Pacific oysters compared to the effects with the non-toxic microalga *Isochrysis galbana*. A sub-chronic exposure feeding on toxic dinoflagellate cells for seven and fourteen days (30×10^3^ cells mL^−1^) showed higher gene expression levels. A significant increase was observed in Cu/Zn-SOD, GST, and LGBP at day 7 and a major increase in GS and CPY450 at day 14. We also observed that oysters fed only with *G. catenatum* (3×10^3^ cells mL^−1^) produced a significant increase on the transcription level than in a mixed diet (3×10^3^ cells mL^−1^ of *G. catenatum*+0.75×10^6^ cells mL^−1^ *I. galbana*) in all the analyzed genes.

**Conclusions:**

Our results provide gene expression data of PST producer dinoflagellate *G. catenatum* toxic effects on *C. gigas*, a commercially important bivalve. Over expressed genes indicate the activation of a potent protective mechanism, whose response depends on both cell concentration and exposure time against these toxic microalgae. Given the importance of dinoflagellate blooms in coastal environments, these results provide a more comprehensive overview of how oysters respond to stress generated by toxic dinoflagellate exposure.

## Introduction

Marine bivalves accumulate toxins during harmful algal bloom events (HABs), acting as principal vectors posing a health hazard to humans who consume them [1], [2]. The Pacific oyster *Crassostrea gigas* may accumulate paralytic shellfish toxins (PSTs), a class of well-known neurotoxins closely associated with HABs, which may be highly toxic and are present over wide coastal areas [2]–[6].

Paralytic shellfish toxins consist of more than 20 structurally related derivatives with dissimilar toxic potencies. Saxitoxin (STX) is the representative compound that has a tricyclic guanidine linked to a hydromethyluracil group [2], [3]. PSTs exert their effect by binding reversibly to voltage-sensitive sodium channels on excitable cell membranes and blocking neurotransmission [1], [3]; *Alexandrium, Pyrodinium*, and *Gymnodinium* genera are the dominant sources of marine PSTs [7]. Consequently, bivalves' feeding physiology in the presence of toxic dinoflagellates is an important research topic especially on areas where HABs frequently occur. Previous studies show that bivalves have variable feeding responses to toxic algae, which may be related to their HAB exposure history [1], [6], [7], toxin bioaccumulation [1], [3], [6], dinoflagellate toxicity [6], [8], exposure time, and bivalve mollusk species [1], [6], [9].

Several bivalve behavioral, physiological, and cell responses to harmful algae have already been described, such as filtration activity reduction [1], [9], pseudo-feces production [9], [10], metabolic rate increase [11], reproductive development anomalies [12], and oxygen consumption changes [13]. Immune system alteration has been reported by phagocytic capacity inhibition and hemocyte adhesion [14]–[16]. Effects of *Alexandrium minutum* exposure caused generalized tissue inflammation, principally of digestive organs, and biochemical alterations, such as amylase activity changes, reactive oxygen species production, and phenol oxidase activities [15]. However, few studies have addressed gene expression changes in *C. gigas* in response to toxic algal exposure or to their toxins. These studies revealed high heat shock protein 70 (Hsp70), cytochrome P450 isoform 356A1 (CYP356A1), and fatty acid binding protein (FABP) expression levels on hemocytes exposed to brevetoxin (PbTx-2) [16]; also, other researchers have reported that level expression of the genes involved in antioxidant defense, detoxification, and stress response (GS, GST, Hsp70, and Cu/Zn-SOD) changed in response to *Prorocentrum lima* (diarrheal toxin producer, DSP) [17].

The aim of this study was to examine whether *C. gigas* exposure to the toxic dinoflagellate *G. catenatum* cells promotes changes in the transcript levels of some genes related to defense mechanisms, stress, and cell detoxification, and whether these changes are correlated with cell dose and/or exposure time. *C. gigas* was exposed to *G. catenatum* cells under experimental conditions: oysters were fed with the dinoflagellate, analyzed for acute (24 h) and sub-chronic (14 days) periods, and compared to those that received an innocuous microalga (*Isochrysis galbana*) as food; two dinoflagellate doses were tested: 3×10^3^ and 30×10^3^ cells mL^−1^. Exposure to dinoflagellate PST-producers (*Alexandrium* and *Gymnodinium* genera) at a cell density from 10^4^ to 10^5^ cells L^−1^ is reported as causative of toxic manifestations in wild mollusks [1], [3], [18].

## Materials and Methods

### Microalgal culture

The planktonic dinoflagellate *Gymnodinium catenatum* strain GCCV6, which produces paralyzing shellfish poisons (PSP) that include dc-saxitoxin (STX), gonyautoxins (GTX2,3), saxitoxin (STX), gonyautoxins (GTX1,4), and neo-saxitoxin (neo-STX), was obtained from the CIBNOR Collection of Marine Dinoflagellates; the strain produces between 25.7–101 pg STXeq cell^−1^ [19], [20]. *G. catenatum* was grown in Fernbach flasks with modified f/2-medium [21]. Culture medium was prepared using seawater (35 psu), filtered with 0.45 μ membrane, and sterilized at 121°C, 15 lb for 20 min. Dinoflagellate cultures were maintained at 24°C±1°C, under light/dark and 150 µmol photons m^−2^ s^−1^ light intensity 12 h∶12 h, and were harvested by centrifugation (2500 *g* 10 min^−1^) during the late exponential growth phase (17–19 days after inoculation) [22] to obtain biomass for oyster assays. For the bioassays, cell density was adjusted using cell-counting data of each culture on Sedgwick-Rafter slides (Microscope Olympus BX41, Tokyo, Japan) after sample fixation with Lugol's solution [23].

*Isochrysis galbana* strain ISG-1, routinely used in aquaculture as food for bivalves [24], was provided by the Live Food Laboratory and used in our work as control diet. Cultures were grown in plastic bags with f/2-medium [21] and maintained at 20°C±1°C under constant illumination, 150 µmol photons m^−2^ s^−1^ light intensity.

### Oysters

Diploid juvenile individuals of Pacific oysters (3±1 mm, 0.022±0.008 g) were obtained from the hatchery “Acuacultura Robles” at Las Botellas (Bahía Magdalena), Baja California Sur, Mexico. Organisms were divided into groups and kept in plastic aquariums (20 L) for acclimation in aerated filtered seawater (1 µm) at 21°C and 34 psu in the Wet Biological Safety Laboratory at CIBNOR for 10 days. The maintenance diet, according to the tables reported for the age [25], consisted of bacteria-free *I. galbana* (ISG-1) at 1.5×10^6^ cells mL^−1^,obtained from the Live Food Laboratory (CIBNOR).

### Experimental design and sample collection

The test-dinoflagellate doses (concentrations) were based on field HAB observations of *G. catenatum* cells [18], [22], [26], [27] and toxic-effect reports of PST producers (*Alexandrium* and *Gymnodinium* genera) [1], [3], [7], [26], [27].

Oysters were exposed to two *G. catenatum* (3 and 30×10^3^ cell mL^−1^) cell suspensions combined with a fixed amount of *I. galbana* (0.75×10^6^ cell mL^−1^. Control diet (toxin-negative) consisted only of *I. galbana* (0.75×10^6^ cell mL^−1^) to identify both normal oyster behavior and microalgal ingest. Also, another control (toxin-positive) was included consisting only of *G. catenatum* diet (3×10^3^ cell mL^−1^) to identify the single effect of the toxic dinoflagellate by ingestion and/or starvation. Groups of 25 oysters (in triplicate) were maintained in 100 mL transparent polypropylene containers with a 1∶1 microalgal mixture in a final volume of 50 mL. Microalgal ration was provided each day as a single dose; aeration was used during feeding experiments to avoid cell sedimentation.

Five organisms of each experimental unit were randomly sampled after 6, 12, and 24 h (acute response) and 7–14 days (sub-chronic response). The sampled organisms corresponding to each sample time were replaced on each experimental unit by oysters exposed in the same experimental conditions (mirror exposure units used for replacement only). Samples were placed on Eppendorf® tubes, then washed with sterile seawater, and finally frozen at −80°C until use.

### Clearance rate (CR)

Feeding rate (clearance rate, CR) was defined as the cleared water volume per unit time. In the bioassay, CR was measured by Coughlan's method [28]. Water-cell samples for cell counting were taken after 24 h of *G. catenatum* exposure, then fixed with Lugol's solution [23] and allowed to stand (at room temperature) for sedimentation and subsequent counting.

### Total RNA preparation and first strand cDNA synthesis

Samples were thawed on ice and total RNA was extracted with TRIzol® Reagent manufacturers' protocol (Life Technologies, Carlsbad, California). Samples (n = 5) were homogenized using a glass pestle; later, two consecutive TRIzol® extractions were done at each sample. RNA quality was verified by visual inspection of 18S and 28S ribosomal RNA bands on agarose-TBE gels, as well as absence of visible genomic DNA contamination. Nucleic acid purity and concentration were determined by spectrophotometry Nanodrop 2000® (Thermo Scientific, Chicago, IL). To ensure complete DNA absence, a direct PCR was done with 1 µL of each RNA preparation using 28S ribosomal specific primers (Table 1) as a no-amplification control. Afterwards, from each verified RNA sample, 0.5 µg were used for cDNA synthesis using The SuperScript™ III First-Strand Synthesis System SuperMix® (Life Technologies, Carlsbad, California). Total RNA was reverse-transcribed by oligo-dT, and resulting cDNA was stored at −80°C until use.

**Table 1:** Primer details of target genes used on real-time PCR analysis and reference genes (*).

Genes	5′-3′ primer sequence		Amplicon size (pb)	Primer efficiency	R^2^	Genbank ref.
	Forward primer	Reverse primer				
Cg-28S*	GGAGTCGGGTTGTTTGAGAATGC	GTTCTTTTCAACTTTCCCTCACGG	114	1.97	0.99	AY632555
Cg-GAPDH*	GTTCAAATATGATTCAACTCACGG	TGGATCCCGTTCGCAATATACG	109	2.00	0.99	AB122066
Cg-GS	CAAACCCCAAAAGAATGCCCTGT	GAAGACCTCACACATCACCAGC	153	1.88	0.97	AJ558239
Cg-GST	GACCCCAGATGACCCTTACCG	CCGAAACAAACTGAGAGAAGACC	71	1.90	0.99	CB617406
Cg-Cu/znSOD	GACAAAATGATTGACTTGGCCGG	CACTCCACAAGCCAATCGTCCG	144	1.85	0.97	AJ496219
Cg-CPY450	ACAGGGACTTCATTGACAGCATG	ATTGTGAAACGAGTACTGTCTACC	151	1.90	0.99	EF645271
Cg- LGBP y β 1–3 glucan	TTGTCCAGTTCTCCCAGCTTCC	GACACTGGAATGGGATGAAGAAC	108	1.95	0.99	CB617438

### Gene expression analysis by Quantitative real-time PCR (qPCR)

A set of five primer pairs was designed based on partial sequences reported for each evaluated gene (Table 1) sharing the following characteristics: primer length from 18 to 20 bp, Tm from 59°C to 61°C, and GC% from 40% to 60%; primer sequences and expected length of each amplicon. Prior to gene-expression quantification, efficiency of each primer pair was determined using the standard curve method [29]. Amplification efficiencies (E) for each primer (reference or target gene) were determined by slope calculation of 4-fold serial dilutions, starting with 80 ng/µL of cDNA and using a fixed fluorescence threshold value of 0.0355.

All qPCR reactions were conducted in triplicate in holding Strip Tubes® (0.1 mL) (Qiagen™), using a Rotor gene 6000 Real-Time PCR detection system® (Qiagen™). A qPCR cocktail-mix was carefully prepared in our laboratory. The mix contained 50 mM MgCl_2_, 2 mM dNTP (each), 0.3 U of Platinum Taq DNA polymerase® (Life Technologies, Carlsbad, California), 0.05 µM of each primer, 20× EvaGreen fluorescent dye® (Biotium™, Hayward, CA), and 3.2 ng/µL of cDNA in 15 µL of final volume per reaction.

Amplification conditions were: 95°C (5 min), 40 cycles of 95°C (60 s), 61°C (30 s), and 72°C (5 s) acquiring fluorescence at 79°C (1 s); finally, a dissociation step from 65°C to 95°C (1°C/s) was done. A melting curve analysis of amplification products was performed at the end of each qRT-PCR reaction to confirm that only one PCR product was amplified and detected.

### Statistical analysis

Data of qRT-PCR were based on CT values. CT is defined as the PCR cycle at which the fluorescence signal crossed a threshold line that was placed in the exponential phase of the amplification curve. The comparative CT method [30] was used to analyze gene expression levels. The CT for the gene target amplification and the CT for the internal controls 28S ribosomal RNA (28S) and glyceraldehyde-3-phosphate dehydrogenase (GAPDH) were determined for each sample. The negative control group was used as the reference sample (calibrator). The expression was quantified through 2-^ΔΔCT^ [31], which represents an n-fold difference relative to the calibrator.

Data were analyzed using two-way ANOVA; significant differences were obtained with the Fisher's multiple test comparison (α = 0.05). All analyses were performed with Statistic 8.0® software (StatSoft, Tulsa, OK). Significant differences were set at *p*<0.05.

## Results

### Crassostrea gigas feeding behavior

Oysters showed immediate changes in their feeding behavior after their contact with *G. catenatum*. After a few minutes of dinoflagellate exposure, clearance rate was significantly reduced (data not shown). During the next two hours, organisms showed a partial valve closure with diet mix of 3×10^3^ cells mL^−1^ and with only *G. catenatum* diet (positive control), but shell valve closure was total in addition to an evident mantle retraction (away from the shell edge) with diet mix of 30×10^3^ cells mL^−1^. However, at 3 h exposure oysters produced pseudo-feces, and only after 4 h exposure did they appear to filtrate normally, principally at 3×10^3^ cells mL^−1^.

**Figure 1:**
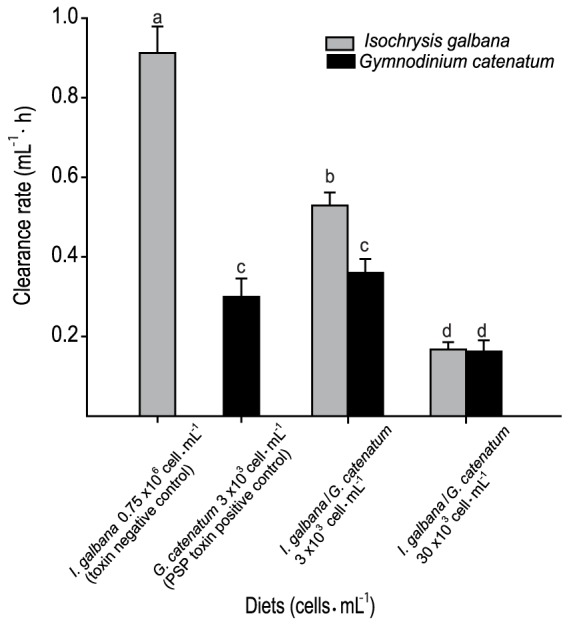
Clearance rate of *Crassostrea gigas* fed *Gymnodinium catenatum* and/or *Isochrysis galbana*.

Clearance rate was significantly different among treatments and negative control (*I. galbana* diet). At 24 hours, CR data of oysters exposed to different experimental conditions are shown in Figure 1. Oysters fed with negative control had a clearance rate of 0.91±0.03, while with specific dinoflagellate, CR was reduced to 0.36±0.03 and 0.16±0.03, with 3×10^3^ cells mL^−1^ and 30×10^3^ cells mL^−1^, respectively. CR showed significant differences (ANOVA, *p*<0.05). It is important to highlight that CR of the positive control (only *G. catenatum* diet) was 0.3±0.05, similar to that of diet mix of 3×10^3^ cells mL^−1^.

Oysters fed with dinoflagellates (positive control and treatments) exhibited a low feeding activity when compared with those fed with non-toxic microalgae (toxin negative control). At 24 h, oysters fed on negative control had completely consumed their food, while those fed with toxic dinoflagellates had not. No deaths occurred under the experimental conditions tested.

### Gene expression analysis in oysters after acute exposure

Toxic dinoflagellate diet effects on *C. gigas* were evaluated by analyzing expression levels of 5 genes that were chosen since they are involved in stress responses (GS), intermediate immune response activation (LGBP), antioxidant defense (Cu/Zn-SOD), and cell detoxification (GST and CPY450).

Quantitative analyses by PCR were performed to examine the expression patterns of each selected gene during acute response. Expression analyses were done using cDNA samples generated from homogenates of five whole spat oysters (one homogenate for each sampled time and experimental replica).

Expression levels of each target gene in the tested experimental conditions (oysters fed on *I.galbana*/*G. catenatum* mixtures) and positive control (oysters fed on *G. catenatum*) were compared to the toxin negative control condition (oysters fed on *I. galbana*) “baseline”. A stability analysis of a set of reference genes (data not published) showed that the most stable gene pair was 28S and GAPDH. No expression difference was observed between unchallenged and challenged oysters. Hence, these genes were used to normalize transcript levels.

**Figure 2:**
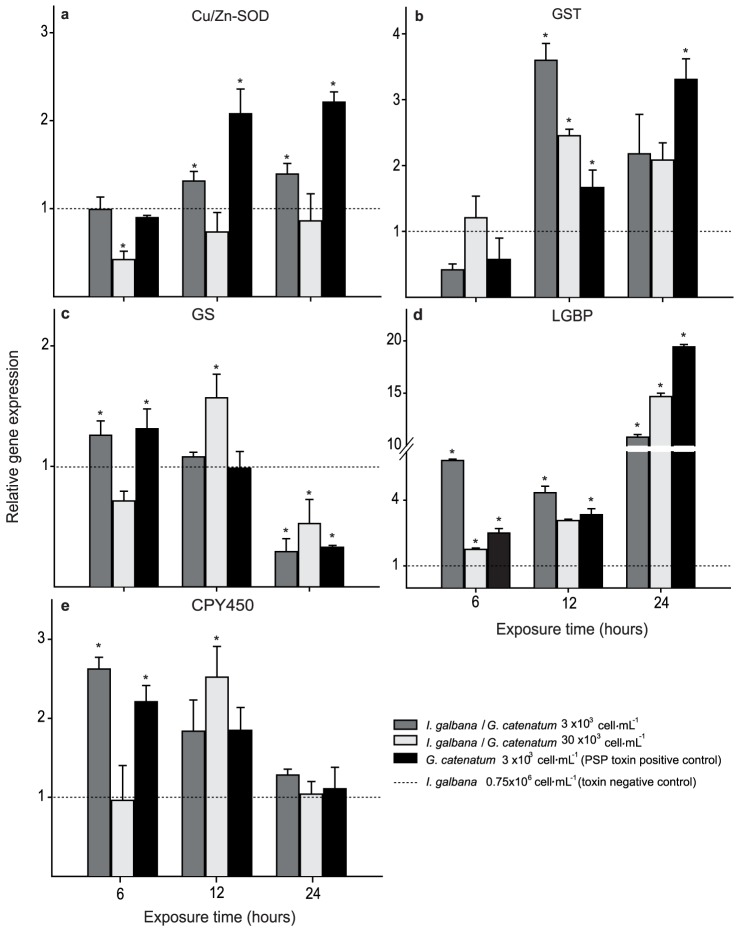
Quantitative expression (qPCR) of *Crassostrea gigas* target genes at acute exposure to *Gymnodinium catenatum* and/or *Isochrysis galbana* cells. Panels: a) Copper/zinc superoxide dismutase, b) Glutathione S transferase, c) Glutamine synthetase, d) Lipopolysaccharide and beta glucan binding protein, and e) Cytochrome p450. Each gene expression is shown as relative expression (2^−ΔΔCt^) with 28S ribosomal and GAPDH as endogenous controls. Asterisk indicates significant differences between treatments and negative control (*p*<0.05 in Fisher's HSD).

On all analyzed genes, changes at transcription level occurred as acute exposure response to toxic dinoflagellates. The expression levels shown by the Cu/Zn-SOD gene were affected in challenged oysters. The transcript level of mixture with 3×10^3^ cells mL^−1^, increased (0.31 fold) above baseline at the first 12 h post challenge, and these levels were maintained for 24 h. In contrast, transcript abundance in the mixture with 30×10^3^ cells mL^−1^ strongly decreased at first hours (0.4 fold below baseline), and it was equal to baseline at 24 h (Fig. 2a). Transcript abundance increased significantly (1.1 fold above baseline) in oysters fed only *G. catenatum* after 12 h.

GST transcript levels were reduced in mixture with 3×10^3^ cells mL^−1^ and in positive control at first 6 h. A significant expression level increase (*p*<0.05, Fig. 2b) was observed in all treatments after 12 h post challenge. The highest transcript level was at 12 h (2.6 fold baseline) in mixture with 3×10^3^ cells mL^−1^ and at 24 h (2.3 fold above baseline) in positive control. Contrary to the behavior of the GST gen, GS showed an increase in expression levels at first 6 h in oysters fed mixture with 3×10^3^ cells mL^−1^ and in those fed with positive control diet (Fig. 2c). However, the expression level of these treatments returned to an expression level equal to that of unchallenged oysters at 12 h, and the transcript level increased significantly (0.6 fold) in oysters fed with 30×10^3^ cells mL^−1^. In all treatments a significant expression level decrease (*p*<0.05) was obtained at 24 h post challenge.

Concerning LGBP, the expression level of challenged oysters increased significantly (*p*<0.05; Fig. 2d) at 6 h post exposure; we observed the increase of 4.8 fold above baseline. At 12 h post challenge transcript levels were maintained from 2.3 to 3.3 fold above baseline. The highest expression level was observed in all treatments at 24 h. We found a higher increase (18.43 fold) in oysters fed only *G. catenatum*.

The transcript levels of CPY450 gene showed an increase during the first 6 hours principally in oysters fed with mixture of 3×10^3^ cells mL^−1^ and positive control reaching values of 1.6 and 1.2 above baseline, respectively, which were maintained until 12 h. At 12 h, oysters fed mixture with 30×10^3^ cells mL^−1^ their expression level increased 1.5 fold (*p*<0.05; Fig. 2e). Transcript levels returned to an expression level equal to that of unchallenged oysters in all treatments at 24 h.

### Gene expression analysis in oysters after sub-chronic exposure

**Figure 3:**
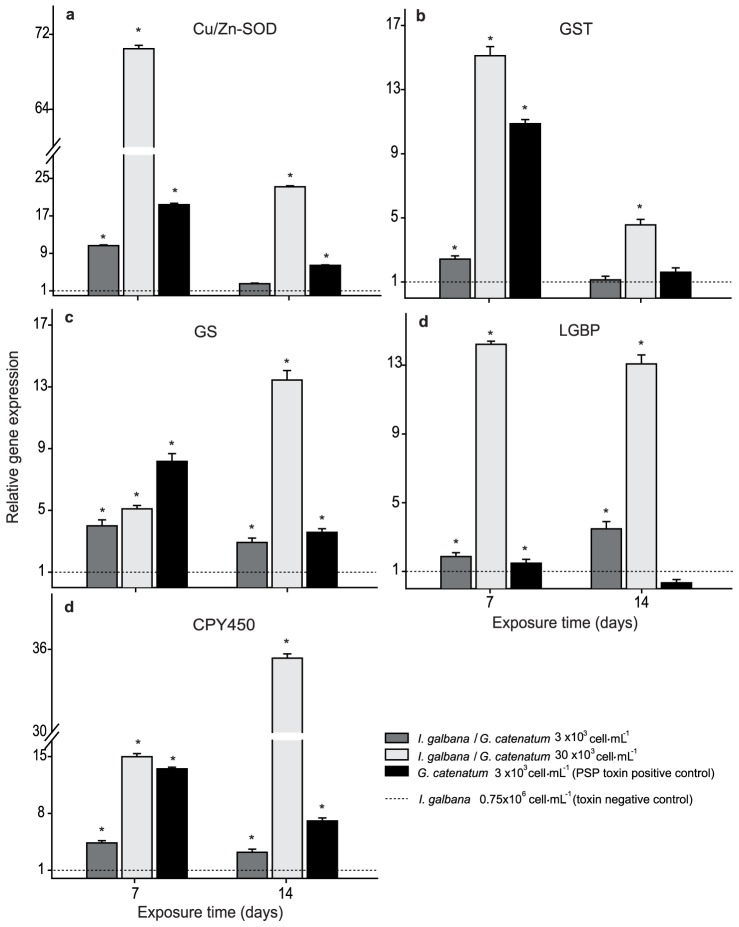
Quantitative expression (qPCR) of *Crassostrea gigas* target genes at sub-chronic exposure to *Gymnodinium catenatum* and/or *Isochrysis galbana* cells. Panels: a) Copper/zinc superoxide dismutase, b) Glutathione S transferase, c) Glutamine synthetase, d) Lipopolysaccharide and beta glucan binding protein, and e) Cytochrome p450. Each gene expression is shown as relative expression (2^−ΔΔCt^) with 28S ribosomal and GAPDH as endogenous controls. Asterisk indicates significant differences between treatments and negative control (*p*<0.05 in Fisher's HSD).

The genes involved in antioxidant defense and cell detoxification (Cu/Zn-SOD and GST) showed a general pattern of major increase at 7 days post challenged and a reduction in transcript abundance at 14 days in all treatments (Fig. 3a). Cu/Zn-SOD transcript abundance increased in oysters fed with mixture 3×10^3^ cells mL^−1^ (10 fold above baseline, *p*<0.05). Also, it strongly increased in oysters fed with mixture 30×10^3^ cells mL^−1^ (71 fold above baseline, *p*<0.05), and finally in oysters fed only *G. catenatum* (18 fold above baseline, *p*<0.05) at 7 days post challenge; these expression levels decreased at 14 days, nonetheless, they remained above baseline expression. GST transcript abundance changed 3 fold in mixture with 3×10^3^ cells mL^−1^, increasing considerably with 30×10^3^ cells mL^−1^ (15 fold) and in positive control (11 fold). Levels also decreased at 14 days (Fig. 3b). We observed a significant increase (*p*<0.05; Fig. 3c) on the expression levels of the GS gen in all treatments (4, 5, and 8 fold) above baseline at 7 days. However, expression level decreased in mixture with 3×10^3^ cells mL^−1^ and positive control but strongly increased in 30×10^3^ cells mL^−1^ (13 fold) at 14 days post challenge.

The LGBP gene showed a very particular expression behavior (Fig. 3d). Differences were related to the amount of toxic cells present at the bioassay system; significant differences (*p*<0.05) were observed mainly in oysters fed with 30×10^3^ cells mL^−1^ at 7 and 14 d (12 fold above baseline). At day 14, this gene remained overexpressed (3 fold) in oysters treated with 3×10^3^ cells mL^−1^, but it was repressed in the positive control.

The CPY450 gene showed a significant increase (*p*<0.05) with 30×10^3^ cells mL^−1^, which was higher than 15 and 35 fold at 7 d and 14 d post challenged, respectively when compared to unchallenged oysters (Fig. 3e). Expression levels in the positive control were higher, 14 fold above baseline at day 7, but we found they decreased to 7 fold at day 14.

The results indicated that the expression levels of these 5 genes in *C. gigas* were affected by the presence of the toxic dinoflagellate, and the magnitude was dependent on time and toxic cell concentration.

## Discussion

As a filter-feeder species, *C. gigas* may accumulate toxins [1], [4], [5], [32] and xenobiotics [33]–[35]; thus toxin presence is one of the major determinants of its sanitary quality. Toxin mechanisms and effects in human and other mammalian species are widely known. However, only recently have researchers begun to study toxic microalgal effects in mollusks (vectors for human toxins), determining they have been mainly physiological [1], [6], [9]. Even less studied are HAB impact effects on filter-feeder organisms, considering shellfish as part of an ecosystem. Thus experiments in this work were designed exposing *C. gigas* to *G. catenatum* cells under experimental conditions to get new information from a different viewpoint. Our goal was to analyze changes occurring in a filter-feeder organism when exposed to toxins through a red tide event (in our case PSP through *G. catenatum*). Finally, duration of the bioassays was designed to determine short and medium term effects (24 h and 14 days), emulating early and developed stages of a red tide up to its exponential phase. Although this study is limited since it does not consider variables such as simultaneous presence of several phytoplankton species, succession phenomena, and decay, we know it is a good starting point for analyzing the effect of marine toxin producers considering *G. catenatum* and *C. gigas* as study models given their ecological, economic, and public health relevance.

Several species of harmful algae have shown previously to affect oysters' and other bivalves' feeding. For example, *C. gigas* reduces its clearance rate when exposed to the toxic dinoflagellates *Alexandrium minutum*, *Alexandrium tamarense*, and *Alexandrium catenella* [11], [12], [36]–[40]. Our results revealed that during the first 2 hours, treated oysters filtered a moderate quantity of toxic dinoflagellates, showing a partial valve closure behavior. On the other hand, our results showed that oysters preferentially remove *I. galbana* over *G. catenatum* from the mixed microalgae offered as food. In oysters fed with a mixture of 3×10^3^ cells mL^−1^ a clear preference for ingesting *I. galbana* was observed but not in the mixture with 30×10^3^ cells mL^−1^ where only 20% of available cells were removed, independently of the microalgal species (*I. galbana* and/or *G. catenatum*).

In all treatments and positive control (only *G. catenatum*) filtration rate was significantly lower than in the negative control, which showed a CR rate of 91% at 24 h. In general, an inverse relationship between CR and toxic cell dose level was observed. No deaths were registered in acute and sub-chronic exposure, which indicate that oysters can cope with adverse effects of toxic cell consumption.

As mentioned briefly before, we observed pseudo-feces formation in treatments with toxic microalgae, finding a direct relationship between the amounts produced with the cell number provided (data not shown). However, in the mixture with 3×10^3^ cells mL^−1^ diet *G. catenatum* dominated in pseudo-feces composition, indicating that oysters selectively remove it over *I. galbana* (Fig. 1). Under a high cell density condition (30×10^3^ cells mL^−1^) oysters' selectivity is lost because both species are cleared from the system in the same proportion, which can be explained under food availability conditions in excess or provoked by the dinoflagellate toxicity.

In the first scenario, pseudo-feces production has been documented as a fundamental pre-ingestion mechanism [5], [8], [10], not only preventing exceeding the animal's ingestion capacity [10], [32], [37], [40] but also facilitating particle selection: less nutritious particles are rejected, thus the quality of ingested material is proportionally improved [5], [36], [37], [40]. However, in our work we demonstrated that the addition of a toxic dinoflagellate as *G. catenatum* in a diet added with the haptophyte *I. galbana* significantly altered *C. gigas*' filtering capacity and pseudo-feces production. Similar behavior was observed in previous studies [40]–[42]. Given the above, it is demonstrated that the oysters have a higher sensitivity to phycotoxin-producing microalgae.

Genomic approaches allowed a better understanding on the biochemical pathways affected by several test-conditions. For this purpose five genes were selected considering they have different physiological roles, and any or all could be a sensitive tool to detect changes when oysters were exposed to toxic dinoflagellates. In general terms, when *C. gigas* was exposed to *G. catenatum*, the observed responses were time-dependent and exhibited a linear dose-response relationship mainly in the sub-chronic period. Therefore, we found statistically significant differences in the interaction dose-exposure-time. Although basal expression levels of the oysters fed on negative control exhibited certain variability, changes were small with short duration over the course of the bioassay. In treatments, changes in transcript levels (dose-time dependent) suggested a direct relationship to toxic algal ingestion. Change magnitude as well as particular effects could be based on a particular gene's susceptibility to any component of the toxic dinoflagellate.

Antioxidant enzymes have been proposed as environmental impact assessment markers due to metals and some organic xenobiotics that generate oxidative stress [43]–[45]. Cu/Zn- SOD is a cytosolic enzyme that has antioxidant properties. Its function is superoxide radical dismutation (O^−2^) to hydrogen peroxide (H_2_O_2_) and oxygen, during oxidative energy processes [43], [44] diminishing destructive oxidative processes in cells.

At 6 hours, transcript levels were equal to those of unchallenged oysters in positive control and mixture with 3×10^3^ cells mL^−1^ but increased after 12 h of exposure. This result might be interpreted that the number of *G. catenatum* cells (3×10^3^ cells mL^−1^) causes a net positive effect on Cu/Zn-SOD expression in spite of an expression adaptation (increase) that balances conditions allowing an apparent homeostasis in the oyster. We also found that following a brief exposure period with 30×10^3^ cells mL^−1^, the Cu/Zn-SOD expression level was mainly suppressed; the harmful effect was obvious because expression repression persisted from the beginning of the experiment to 24 h, recovering slowly.

However, the trend observed was major under sub-chronic exposure (Fig. 3a) where it revealed an increase in transcript synthesis, finding significantly higher values in 30×10^3^ cells mL^−1^ and in the positive control when compared with mixture of 3×10^3^ cells mL^−1^. In the same figure, it draws our attention that in the positive control Cu/Zn-SOD expression level is higher, independently of exposure time but even higher at 7 days. The difference between these two experimental conditions is the presence of *I. galbana* in the diet, which somehow mitigates damage in the oyster, decreasing the magnitude of the impact.

Recent evidence showed that Cu/Zn-SOD are involved in cell response to many environmental and physiological stressors, which include studies in *C. gigas*, mainly after heavy-metal exposure [45], [46], thermal [47], summer mortality [48], pesticide exposure [34], [35], [49], toxic dinoflagellate *P. lima* exposure [17], finding that transcript levels increased after stress challenges. The Cu/Zn-SOD overexpression is indicative of oxidative stress generated by ROS in oysters, which is the strategy to compensate damage at cellular level, increasing in transcription and then increasing mortality resistance. Therefore, a stronger correlation has been observed between phycotoxins and oxidative damage [17], [50].

Glutathione S-transferase (GST) belongs to a large superfamily of multi-functional enzymes involved in cellular detoxification by catalyzing glutathione (GSH) conjugation to electrophilic compounds with a wide range of endogenous and xenobiotic agents, including environmental toxins and oxidative stress products [51]–[53]. The GST gene transcript levels showed significant differences between oysters exposed to *G. catenatum* and *I. galbana*, so presumably it is regulated and involved in the oysters' metabolism of PST toxin management. In our research we also found that GST expression was low at 6 h in all treatments; however, GST was overexpressed after 12 h in challenged oysters. An early response of *C. gigas* to the presence of *G. catenatum* has been documented and also shown in our study as a decrease in feed through valve closure [41] together with palatability of food as a mechanism of tissue damage resistance [5], [36], [37]. In a sub-chronic exposure, the highest overexpression was found on day 7 (mixture of 30×10^3^ cells mL^−1^ and positive control). A GST overexpression was observed in *C. gigas* exposed to contaminants [53] under sub-chronic exposure as in our work.

The biochemical adaptations that involve a gene response against xenobiotics, as the GST activity, could partly explain the consumers' reluctance to food chemical threats and provide information about enzymatic mechanisms underlying foraging decisions [45], [54], [55]. GST induction with an allelochemical diet can serve as an additional adjustment mechanism of protection against toxicity [54], [55]; this phenomenon may be present in our study system (oyster-toxic dinoflagellate).

The differences on GST transcript level among treatments could be due to detoxification processes normally associated with stress [44], [50], [51], explaining the high GST expression observed in oysters exposed to toxic cells. Moreover, it has been found that oysters have high toxic accumulation on tissues after seven days of exposure [1], [9], which agrees with our observations of increased GST transcripts on exposed oysters during similar time periods; these results contribute to a better understanding of the GST role and the presence of resistance mechanisms towards a toxic dinoflagellate diet. Furthermore, a recurrent GST up-regulation could be associated to an increase of the animal's resistance to stressors, including toxic compounds [54].

Glutamine synthetase (GS) is an enzyme that plays a central role in nitrogen metabolism by catalyzing the chemical reaction of ammonium to glutamate and glutamine formation; it detoxifies ammonia and conveys nitrogen towards urea, amino acid, and nucleotide biosynthesis [56]. In our work a high GS expression level was found in mixture of 3×10^3^ cells mL^−1^ and positive control on dinoflagellate-exposed oysters in regard to control, but the highest increase was at 12 h of exposure in mixture of 30×10^3^ cells mL^−1^. The harmful effect of toxic cell presence on oysters was obvious because of expression repression at 24 h. These data suggest that the only presence of *G. catenatum* affects the oyster's metabolism even in short exposure periods. Likewise, GS expression increase was highly regulated by dinoflagellate cell concentration since responses were time-dependent and exhibited a positive linear dose-response relationship under sub-chronic exposure.

Several studies have found that the GS gene is up regulated in oysters exposed to hydrocarbons [53], hypoxia [57], pesticides [34], [35], [49], [53], or to toxic dinoflagellate *Prorocentrum lima* [17], playing an important role in the organisms' resistance to these stressors. Some studies suggest that glutamine produced by GS is required in the formation of amino acids, purines, and pyrimidines that are essential on protein synthesis [56]. Thus, in our research the increase of GS transcript abundances observed on exposed oysters could reflect an increase in protein synthesis, essential for regulating homeostasis in challenged oysters to dinoflagellates.

The lipopolysaccharide and beta-1, 3-glucan binding protein (LGBP) plays a crucial role in invertebrates' innate immune response [58]. The binding of lipopolysaccharide and/or β-1, 3-glucan to LGBP activates the pro-phenoloxidase cascade, which is apparently a general response of *C. gigas* to stress exposure whatever its abiotic or biotic nature is [58]–[60]. The host defense mechanism can be divided in three essential steps: detecting infecting microorganisms, activating intracellular signaling pathways, and triggering the effector mechanism [58]. The LGBP is an acute phase protein that plays a critical role in host-parasite interactions, essential in the overall response of invertebrates' innate immune system.

Our results indicate a differential response on LGBP related to the tested treatment and exposure time because an overexpression was observed both in the beginning of the bioassay and under sub-chronic exposure (in oysters fed with 30×10^3^ cells mL^−1^ for 14 days; Fig. 3d). In our study, the protective immune response activation on oysters exposed to *G. catenatum* suggests that oysters perceive this dinoflagellate as a non-owned element that could function as a potential invader rather than experiencing physiological impairment due to a chemical toxin in *G. catenatum*; it is noteworthy that oysters did not have the same response in the presence of *I. galbana*.

Although many studies have assessed the effects of harmful algae and their toxins upon bivalves' immune systems, biological interactions between them are poorly understood, especially at molecular level. This gene was previously identified in up regulated SSH libraries from *C. gigas* exposed to hydrocarbons [35] and parasites [61]. Our report provides the first evidence that exposure to *G. catenatum* toxic cells temporarily increases LBGP gene expression and could play a critical role on *C. gigas-G. catenatum* interaction.

The cytochrome P450 (CYP450) supergene family is a large and diverse group of enzymes that generally constitute the first enzymatic defense against foreign compounds. It is characterized as one of the major phase I-type class of detoxification enzymes found in living organisms. These enzymes metabolize a wide variety of substrates, such as fatty acids, hormones, and xenobiotics [62]. Alteration on both CYP450 and glutathione S-transferase has been found in marine organisms after exposure to allelochemical gorgonian corals [54] and to many typical pollutants that are continuously released into the environment: polycyclic aromatic hydrocarbons, heavy metals (e.g. Pb and Cu), and endocrine disruptor chemicals [63], [64]

Overexpression patterns of this important detoxification enzyme were found in sub-chronic exposure; these data suggest a possible role of CPY450 in protecting oysters exposed to toxic cells; even more, CPY450 expression changes were dependent on tested cell concentration. On the other hand, it is well known that bivalves accumulate and metabolize toxins, resulting in conjugated, oxidized, reduced, and hydrolyzed metabolites [1], [65]. Nevertheless, PST biotransformation associated to cytochrome P450 activity has not been described in *C. gigas* yet; thus the observed increase on the CYP450's transcript level found in our work opens the opportunity to study if this enzyme is involved and how in this association.

In our work, we have demonstrated the toxic effect caused by *G. catenatum* using whole cells. It is important to consider that metabolic byproducts other than toxins may contribute to the effects observed by affecting cell membranes or particular organ systems which in sum could affect animal health and ultimately cause disease. Additionally, we provide here the first evidence that exposure to toxic *G. catenatum* cells generates an increase on the expression levels of genes encoding proteins involved in antioxidant stress, detoxification, stress response, and immune defense. We have demonstrated that a low cell number of *G. catenatum* and a short exposure time is enough for *C. gigas* to inhibit or to overexpress these genes, and that a sub-chronic exposure induces major changes in expression profiles. Therefore, the stress induced by the presence of *G. catenatum* probably increases oysters' susceptibility to invasion of opportunistic pathogens, which occurs in natural events since oyster populations die from secondary infections. Further studies can provide further understanding on how *G. catenatum* toxins activate/deactivate oysters' defense systems.

## Conclusions

Toxic dinoflagellate ingestion by oysters causes stress, according to physiological alterations in feeding behavior, antioxidant enzyme modulation of mRNA expression, and the immune system in a way that it depends directly on dose and exposure time. A *G. catenatum* diet appears to alter the homeostasis of oysters' stress genes temporarily, causing diminished health and thus an increase in disease susceptibility. These findings aid in understanding oysters' genomic response to toxic dinoflagellates, making it clear that molecular biology and biochemistry approaches are fundamental tools to identify genes/proteins that have a major role in the toxicity process, and thus in better understanding those impacts.

## References

[1] Bricelj VM, Shumway SE (1998) Paralytic shellfish toxins in bivalve mollusks: occurrence, transfer kinetics and biotransformation. Rev Fish Sci 6: 315–383.

[2] Lehane L (2000) Paralytic shellfish poisoning: a review. National Office of Animal and Plant Health, Agriculture, Fisheries and Forestry: Canberra, Australia. 56 p

[3] Kodama M (2010) Paralytic Shellfish Poisoning Toxins: Biochemistry and Origin. ABSM 3: 1–38.

[4] Lassus P, Baron R, Garen P, Truquet P, Masselin P, et al (2004) Paralytic shellfish poison outbreaks in the Penzé estuary: Environmental factors affecting toxin uptake in the oyster, *Crassostrea gigas* . Aquat Living Resour 214: 207–214.

[5] Bardouil M, Bohec M, Cormerais M, Bougrier S, Lassus P (1993) Experimental study of the effects of a toxic microalgal diet on feeding of the oyster *Crassostrea gigas* Thunberg. J Shellfish Res 12: 417–422.

[6] Landsberg JH (2002) The effects of harmful algal blooms on aquatic organisms. Rev Fish Sci 10: 113–390.

[7] Van Dolah FM (2000) Marine algal toxins: origins, health effects, and their increased occurrence. EHP 108: 133–141. 10698729 10.1289/ehp.00108s1133 PMC1637787

[8] Bricelj VM, Lee JH, Cembella AD, Anderson DM (1991) Influence of dinoflagellate cell toxicity on uptake and loss of paralytic shellfish toxins in the northern quahog, *Mercenaria mercenaria* . Mar Ecol Prog Ser 74: 33–46.

[9] Shumway SE (1990) A review of the effects of algal blooms on shellfish and aquaculture. J World Aquacul Soc 2: 65–104.

[10] Alexander JA, Stoecker DK, Meritt DW, Alexander ST, Johns D, et al (2008) Differential production of feces and pseudo-feces by the oyster *Crassostrea ariakensis* when exposed to diets containing harmful dinoflagellate and raphidophyte species. J Shellfish Res 27 (3) 567–579.

[11] Li SC, Wang WX, Hsieh DPF (2002) Effects of toxic dinoflagellate *Alexandrium tamarense* on the energy budgets and growth of two marine bivalves. Mar Environ Res 53: 145–160. 11829010 10.1016/s0141-1136(01)00117-9

[12] Haberkorn H, Lambert C, Le Goïc N, Moal J, Suquet M, et al (2010) Effects of *Alexandrium minutum* exposure on nutrition-related processes and reproductive output in oysters *Crassostrea gigas* . Harmful Algae 9: 427–439.

[13] Gainey LF, Shumway SE (1988) A compendium of the responses of bivalve mollusks to toxic dinoflagellates. J Shellfish Res 7: 623–628.

[14] Hégaret H, Wikfors GH, Soudant P, Lambert C, Shumway SE, et al (2007) Minimal apparent effect on oyster hemocytes. Fish & shellfish immunology 152: 441–447.

[15] Hégaret H, Brokordt KB, Gaymer CF, Lohrmann KB, García C, et al (2012) Effects of the toxic dinoflagellate *Alexandrium catenella* on histopathogical and escape responses of the northern scallop *Argopecten purpuratus* . Harmful Algae 18: 74–83.

[16] Mello DF, de Oliveira ES, Vieira RC, Simoes E, Trevisan R, et al (2012) Cellular and transcriptional responses of *Crassostrea gigas* hemocytes exposed in vitro to brevetoxin (PbTx-2). Marine drugs 10: 583–597. 22611355 10.3390/md10030583 PMC3347016

[17] Romero-Geraldo RJ, Hernández-Saavedra NY (2012) Stress gene expression in *Crassostrea gigas* (Thunberg, 1793) in response to experimental exposure to the toxic dinoflagellate *Prorocentrum lima* (Ehrenberg) Dodge, 1975. Aquac Res 1–11 doi:10.1111/are.12100

[18] Hernández-Sandoval F, López-Cortés DJ, Band-Schmidt C, Gárate-Lizárraga I, Núñez-Vásquez E (2009) Paralytic toxins in bivalve mollusks during a proliferation of *Gymodinium catenatum* Graham in Bahía de La Paz, Mexico. Hidrobiológica 19: 245–256.

[19] Pérez-Linares J, Ochoa JL, Gago-Martínez A (2008) Effect of PSP toxins in white leg shrimp *Litopenaeus vannamei* Boone, 1931. JFS 73: 69–73. 10.1111/j.1750-3841.2008.00710.x 18460148

[20] Pérez-Linares J, Ochoa JL, Gago-Martínez A (2009) Retention and tissue damage of PSP and NSP toxins in shrimp: Is cultured shrimp a potential vector of toxins to human population. Toxicon 53: 185–195. 19028514 10.1016/j.toxicon.2008.10.022

[21] Guillard RLL, Ryther RH (1975) Culture of phytoplankton for feeding marine invertebrates. In Smith, W.L. and M.M., Chalg, (Eds) Culture of marine invertebrate animals, Plenum Press, New York. pp. 29–60.

[22] Band-Schmitd CJ, Bustillo-Guzmán J, Gárate-Lizárrga I, Lechuga-Devéze CH, Reinhart K, et al (2005) Paralytic shellfish toxin profile in strains of dinoflagellate *Gymnodinium catenatum* Graham and the scallop *Argopecten ventricosus* G-B. Sowerby II from Bahia Concepción. Gulf of California México. Harmful Algae 4: 21–31.

[23] Gifford DJ, Caron DA (2000) Sampling, preservation, enumeration and biomass of marine protozooplankton. In: Harris RP, et al. (Eds) ICES Zooplankton methodology manual. Academic Press, London. pp. 193–221.

[24] Brown MR, Jeffrey SW, Volkman JK, Dunstan GA (1997) Nutritional properties of microalgae for mariculture. Aquaculture 151: 315–331.

[25] Helm MM, Bourne N, Lovatelli A (2004) Hatchery culture of bivalves. A practical manual. FAO Fisheries Technical Paper. 177 p.

[26] Gárate-Lizárraga I, Bustillos-Guzmán J, Alonso-Rodríguez R, Luckas B (2004) Comparative paralytic shellfish toxin profiles in two marine bivalves during outbreaks of *Gymnodinium catenatum* (Dinophyceae) in the Gulf of California. Mar Pollut Bull 48: 397–402. 14972594 10.1016/j.marpolbul.2003.10.032

[27] Band-Schmidt CJ, Bustillos-Guzmán JJ, López-Cortés DJ, Gárate-Lizárraga I, Núñez-Vázquez EJ (2010) Ecological and physiological studies of *Gymnodinium catenatum* in the Mexican Pacific: a review. Marine drugs 8: 1935–1961. 20631876 10.3390/md8061935 PMC2901831

[28] Coughlan J (1969) The estimation of filtering rate from the clearance of suspensions. Mar Biol 2: 356–358.

[29] Bustin SA (2000) Absolute quantification of mRNA using real-time reverse transcription polymerase chain reaction assays. J Mol Endocrinol 169–193. 11013345 10.1677/jme.0.0250169

[30] Vandesompele J, De Preter K, Pattyn F, Poppe B, Van Roy N, et al (2002) Accurate normalization of real-time quantitative RT-PCR data by geometric averaging of multiple internal control genes. Genome Biol 3: 1–12. 10.1186/gb-2002-3-7-research0034 PMC126239 12184808

[31] Livak KJ, Schmittgen TD (2001) Analysis of relative gene expression data using real-time quantitative PCR and the 2(-Delta Delta C(T)) Method. Methods (San Diego, California) 25: 402–408. 10.1006/meth.2001.1262 11846609

[32] Bougrier S (2003) Paralytic shellfish poison accumulation yields and feeding time activity in the Pacific oyster (*Crassostrea gigas*) and king scallop (*Pecten maximus*). Aquat Living Resour 16: 347–352.

[33] Gagnaire B, Thomas-Guyon H, Burgeot T, Renault T (2006) Pollutant effects on Pacific oyster, *Crassostrea gigas* (Thunberg, 1793), hemocytes: Screening of 23 molecules using flow cytometry. Cell Biol Toxicol 22: 1–14. 16463015 10.1007/s10565-006-0011-6

[34] Collin H, Meistertzheim AL, Moraga D, Boutet I (2010) Response of the Pacific oyster *Crassostrea gigas* (Thunberg 1793), to pesticide exposure under experimental conditions. J Exp biol 213: 4010–4017. 21075942 10.1242/jeb.048033

[35] Boutet I, Tanguy A, Moraga D (2004) Response of the Pacific oyster *Crassostrea gigas* to hydrocarbon contamination under experimental conditions. Gene 329: 147–157. 15033537 10.1016/j.gene.2003.12.027

[36] Lassus P, Wildish D, Bardouil M, Martin JL, Bohec M, et al. (1996) Ecophysiological study of toxic *Alexandrium spp* effects on the oyster *Crassostrea gigas*. In: Yasumoto, T., Oshima, Y., Fukuyo, Y. (Eds.), Harmful and Toxic Algal Blooms. IOC-UNESCO Publ. pp. 409–412.

[37] Wildish D, Lassus P, Martin J, Saulnier A, Bardouil M (1998) Effect of the PSP causing dinoflagellate, *Alexandrium* spp. on the initial feeding response of *Crassostrea gigas* . Aquat Living Resour 11: 35–43.

[38] Laabir M, Amzil Z, Lassus P, Masseret E, Tapilatu Y, et al (2007) Viability, growth and toxicity of *Alexandrium catenella* and *Alexandrium minutum* (Dinophyceae) following ingestion and gut passage in the oyster *Crassostrea gigas* . Aquat Living Resour 57: 51–57.

[39] Lassus P, Amzil Z, Baron R, Séchet V, Barillé L, et al (2007) Modelling the accumulation of PSP toxins in Thau Lagoon oysters (*Crassostrea gigas*) from trials using mixed cultures of *Alexandrium catenella* and *Thalassiosira weissflogii* . Aquat Living Resour 67: 59–67.

[40] Haberkorn H, Lambert C, Le Goïc N, Guéguen M, Moal J, et al (2010) Effects of *Alexandrium minutum* exposure upon physiological and hematological variables of diploid and triploid oysters, *Crassostrea gigas* . Aquatic toxicol 97: 96–108. 10.1016/j.aquatox.2009.12.006 20045204

[41] Estrada NA, Lagos N, García C, Maeda-Martinez A, Ascencio F (2007) Effects of the toxic dinoflagellate *Gymnodinium catenatum* on uptake and fate of paralytic shellfish poisons in the Pacific giant lions-paw scallop *Nodipecten subnodosus* . Mar Biol 151: 1205–1214.

[42] Navarro JM, Contreras AM (2010) An integrative response by *Mytillus chilensis* to the toxic dinoflagellate *Alexandrium catenella* . Marine Biology 157: 1967–1974.

[43] Halliwell B, Gutteridge JMC (1999) Free radicals in biology and medicine. New York: Oxford Univ. Press. 936 p

[44] Lesser PM (2006) Oxidative stress in marine environments: Biochemistry and Physiological Ecology. Annual Review of Physiology 68: 253–78. 10.1146/annurev.physiol.68.040104.110001 16460273

[45] Tomanek L (2011) Environmental proteomics: changes in the proteome of marine organisms in response to environmental stress, pollutants, infection, symbiosis, and development. Annu Rev Mar Sci 3: 373–399. 10.1146/annurev-marine-120709-142729 21329210

[46] Jenny MJ, Ringwood AH, Schey K, Warr GW, Chapman RW (2004) Diversity of metallothioneins in the American oyster, *Crassostrea virginica*, revealed by transcriptomic and proteomic approaches. Eur J Biochem 271: 1702–1712. 15096209 10.1111/j.1432-1033.2004.04071.x

[47] Meistertzheim A, Tanguy A, Moraga D (2007) Identification of differentially expressed genes of the Pacific oyster *Crassostrea gigas* exposed to prolonged thermal stress. FEBS J 274: 6392–6402. 18005253 10.1111/j.1742-4658.2007.06156.x

[48] Huvet A, Herpin A, Dégremont L, Labreuche Y, Samain JF, et al (2004) The identification of genes from the oyster *Crassostrea gigas* that are differentially expressed in progeny exhibiting opposed susceptibility to summer mortality. Gene 343: 211–220. 15563847 10.1016/j.gene.2004.09.008

[49] Tanguy A, Boutet I, Laroche J, Moraga D (2005) Molecular identification and expression study of differentially regulated genes in the Pacific oyster *Crassostrea gigas* in response to pesticide exposure. FEBS J 272: 390–403. 15654877 10.1111/j.1742-4658.2004.04479.x

[50] Estrada N, Campa-Córdova A, Luna A, Ascencio F (2007) Effects of the toxic dinoflagellate, *Gymnodinium catenatum* on hydrolytic and antioxidant enzymes, in tissues of the giant lions-paw scallop *Nodipecten subnodosus* . Comp Biochem Phys C 146: 502–510. 10.1016/j.cbpc.2007.06.003 17613278

[51] Manduzio H, Rocher B, Durand F, Galap C, Leboulenger F (2005) The point about oxidative stress in mollusks. ISJ 2: 91–104.

[52] Campa-Córdova AI, González-Ocampo H, Luna-González A, Mazón-Suástegui JM, Ascencio F (2009) Growth, survival, and superoxide dismutase activity in juvenile *Crassostrea corteziensis* (Hertlein, 1951) treated with probiotics. Journal of Aquaculture in the Tropics 19: 151–157.

[53] Boutet I, Tanguy A, Moraga D (2004) Characterization and expression of four mRNA sequences encoding glutathione S-transferases pi, mu, omega and sigma classes in the Pacific oyster. Mar Biol 146: 53–64.

[54] Vrolijk NH, Targett NM (1992) Biotransformation enzymes in *Cyphoma gibbosum* (Gastropoda: Ovulidae): implications for detoxification of gorgonian allelochernicals. Mar Environ Res 88: 237–246.

[55] Whalen EK, Morin D, Lin YC, Tjeerdema SR, Goldstone VJ (2008) Proteomic identification, cDNA cloning and enzymatic activity of glutathione S-transferases from the generalist marine gastropod, *Cyphoma gibbosum* . Arch Biochem Biophys 478: 7–17. 18671936 10.1016/j.abb.2008.07.007

[56] Keppler CJ (2007) Effects of ammonia on cellular biomarker responses in oysters (*Crassostrea virginica*). Ecol Eng 63–66. 10.1007/s00128-007-9007-z 17333422

[57] David E, Tanguy A, Pichavant K, Moraga D (2005) Response of the Pacific oyster *Crassostrea gigas* to hypoxia exposure under experimental conditions. FEBS J 272: 5635–5652. 16262701 10.1111/j.1742-4658.2005.04960.x

[58] Girón-Pérez MI (2010) Relationships between innate immunity in bivalve mollusks and environmental pollution. ISJ 7: 149–156.

[59] Guéguen Y, Cadoret JP, Flament D, Barreau-Roumiguiére C, Girardot AL, et al (2003) Immune gene discovery by expressed sequence tags generated from hemocytes of the bacteria challenged oyster, *Crassostrea gigas* . Gene 303: 139–145. 12559575 10.1016/s0378-1119(02)01149-6

[60] Montagnani C, Avarre JC, de Lorgeril J, Quiquand M, Boulo V, et al (2007) First evidence of the activation of Cg-timp, an immune response component of Pacific oysters, through a damage-associated molecular pattern pathway. Dev Comp Immunol 31: 1–11. 16793134 10.1016/j.dci.2006.04.002

[61] Tanguy A, Ximing G, Ford SE (2004) Discovery of genes expressed in response to *Perkinsus marinus* challenge in Eastern (*Crassostrea virginica*) and Pacific (C. gigas) oysters. Gene 338: 121–131. 15302413 10.1016/j.gene.2004.05.019

[62] Ziegler DM (1993) Recent studies on structure and function of multisubstrate flavin-containing monooxygenases. Annu Rev Pharmacol Toxicol 33: 179–199. 8494339 10.1146/annurev.pa.33.040193.001143

[63] Tanguy A, Boutet I, Bonhomme F, Boudry P, Moraga D (2002) Polymorphism of metallothionein genes in the Pacific oyster *Crassostrea gigas* as a biomarker of response to metal exposure. Biomarkers 7: 439–450. 12581480 10.1080/13547500210157531

[64] Boutet I, Tanguy A, Moraga D (2004) Molecular identification and expression of two non-P450 enzymes, monoamine oxidase A and flavin-containing monooxygenase 2, involved in phase I of xenobiotic biotransformation in the Pacific oyster, *Crassostrea gigas* . Biochimica et Bophysica Acta 1679: 29–36. 10.1016/j.bbaexp.2004.04.001 15245914

[65] Choi MC, Hsieh DPH, Lam PKS, Wang WX (2003) Field depuration and biotransformation of paralytic shellfish toxins in scallop *Chlamys nobilis* and green-lipped mussel *Perna viridis* . Mar Biol 143: 927–934.

